# A Systematic Review of Estrogen Modulators as Augmentation to Antipsychotics for the Treatment of Post- and Perimenopausal Psychosis

**DOI:** 10.1192/bjo.2024.180

**Published:** 2024-08-01

**Authors:** Cassidy Keen, Athanasios Hassoulas, Jill Richardson

**Affiliations:** 1 Brunel University London, London, United Kingdom; 2 Cardiff University, Cardiff, United Kingdom

## Abstract

**Aims:**

To investigate if estrogen agents as an adjunct to antipsychotic medication are effective at treating psychosis in post-and perimenopausal females.

**Methods:**

A digital search focusing on controlled clinical trials was conducted. Studies were assessed for quality using the Cochrane Risk of Bias tool and GRADE system. The Joanna Briggs Institute (JBI) tools were used to critically appraise articles. The total Positive and Negative Symptom Scale (PANSS) scores were synthesised using a meta-analysis.

**Results:**

Of the studies obtained (n = 11), two used estrogen HT as an augmentation agent, and nine used the SERM Raloxifene. Quality review and critical appraisal found inconsistencies in data and publication bias favouring trials that include Raloxifene. Meta-analysis results indicate Raloxifene plus antipsychotic did perform better than placebo [Std diff in means total = 0.340 (95% CI) p = 0.001] with a small effect size (g = 0.3392).

**Conclusion:**

Though research appears promising, recommendations for the use of estrogen agent augmentation cannot be made at this time as more clinical trials that include a diverse range of treatments are needed.

